# The Human Right to Breathe Clean Air

**DOI:** 10.5334/aogh.2646

**Published:** 2019-12-16

**Authors:** David R. Boyd

**Affiliations:** 1 Institute for Resources, Environment and Sustainability, University of British Columbia, CA

## Abstract

Five national academies of science and medicine—from Brazil, Germany, South Africa, and the United States—issued a powerful statement about air pollution’s immense impacts on public health. The statement concluded that the evidence linking air pollution and adverse health effects is unequivocal, the costs are enormous and yet the problem is preventable. However it is insufficient to treat clean air as a policy objective. It must be regarded as a fundamental human right, related to the rights of life, health, and a safe, clean, healthy and sustainable environment. The human rights perspective changes everything, because governments have clear, legally enforceable obligations to respect, protect, and fulfill human rights.

Dirty air is causing a global public health crisis. Every minute, a child dies of illness caused by air pollution. Every minute, ten adults die, prematurely, because of dirty air inhaled during their lifetime. The total, at least five million deaths annually, is larger than the annual total of deaths caused by war, murder, car accidents, plane crashes, malaria, tuberculosis, HIV/AIDS, and Ebola combined [1].

Over ninety percent of the world’s population lives in regions where air pollution exceeds World Health Organization standards. The very worst air quality, somewhat surprisingly, is found in homes where solid fuels are used for cooking and heating. Women and children in particular are exposed to air pollution in the supposed safety of their own homes at levels far higher than found in even the world’s most smog-ravaged cities.

In response to this crisis, five of the world’s leading academies of science and medicine—from Brazil, Germany, South Africa, and the United States—issued a compelling statement about air pollution’s terrible toll in June 2019. These experts made three key points.

First, the scientific evidence about the impacts of air pollution on human health is unequivocal. Breathing dirty air causes respiratory illness, heart disease, stroke, lung cancer, negative birth outcomes, and a range of other problems.

Second, the economic costs inflicted by poor air quality, especially in low and middle-income countries are enormous. Children missing school, adults missing work, health care costs and the value of lives lost add up to trillions of dollars annually.

Third, air pollution is preventable through the application of strong policies and investments in clean technologies. These solutions are well established and the benefits of their implementation vastly exceed their costs [2].

The scientific and medical academies concluded with a plea to policymakers and businesses: urgent action is needed.

For decades, governments have treated air pollution as an environmental issue. Recently it has started being treated as a health issue. Both of these approaches identify cleaner air as a policy goal. But policy goals are inadequate because they are undermined by flexibility, discretion, and the absence of accountability.

However, air pollution is also a human rights issue. Air pollution on today’s scale clearly violates the rights to life and health, the rights of the child, and the right to live in a safe, clean, healthy and sustainable environment.

The human rights perspective changes everything, because governments have clear, legally enforceable obligations to respect, protect, and fulfill human rights.

Clean air and clean water are both essential to human health and well-being. In 2010, the United Nations General Assembly passed a groundbreaking resolution, recognizing that access to clean water is a basic human right. Around the world, genuine progress is being made in providing clean water to tens of millions of people every year [3].

Remarkably, no similar UN resolution on the right to breathe clean air, or the right to live in a healthy environment, which surely includes clean air, has ever been passed. Surely the time has come.

What consequences would flow from recognizing that everyone, everywhere has the right to breathe clean air?

In a recent report to the Human Rights Council, I set forth seven key steps that states need to take in order to fulfill their legal obligation to protect human rights from air pollution [4]. These include: establishing air quality monitoring networks; quantifying the main sources of air pollution; engaging and informing the public; enacting strong laws, regulations, and air quality standards; developing a national action plan to achieve the standards; allocating adequate resources to implement the plan; and evaluating progress to determine if there is any necessity for stronger actions.

There is irrefutable evidence that stronger laws, standards and policies, combined with large investments in clean technologies can make an enormous difference. As an added bonus, the changes needed to reduce air pollution are often exactly the same changes needed to reduce the greenhouse gas emissions causing climate change. And the vulnerable people currently suffering from the worst air quality should be the primary beneficiaries of a rights-based approach.

Because of the extremely high exposures caused by cooking with solid fuels, making the switch to clean cooking stoves and fuels must be a global priority. India and Indonesia have made impressive progress by providing free LPG (liquefied petroleum gas) stoves and subsidized fuel to over one hundred million poor families. These stoves save cooking time, reduce the burden of gathering fuels such as firewood, and dramatically reduce pollution, delivering major health benefits. This is probably the only situation in the world where it makes sense to subsidize the increased use of fossil fuels.

The World Bank estimated that switching all remaining households to clean stoves and clean fuels by 2030, consistent with the UN Sustainable Development Goals, would require an investment of approximately $US 5 billion per year. In light of the health benefits, time savings and associated economic opportunities for women, quality of life improvements, reduced air pollution and greenhouse gas emissions, and reduced pressure on forests (for firewood), this is a fantastic investment. This sum also fits easily within the $US 100 billion in annual financial assistance that wealthy nations have committed to mobilize for low-income countries to address the challenges of climate change.

Other proven solutions include clean air legislation, replacing coal-fired electricity with renewables, emphasizing walking and cycling in cities, electrifying public transit, ending fossil fuel subsidies (except as indicated above), improving waste management, and helping farmers shift to cleaner practices. Implementing these actions on a widespread and rapid scale would improve air quality, respond to the global climate emergency, and produce immense health, environmental, social, and economic benefits.

There is no room left for equivocating, no time left for debate. Clean air is not an optional policy objective. It’s a fundamental human right.

Everyone needs to breathe clean air. That billions of people today are breathing dirty, deadly air constitutes a global health, environmental and human rights crisis. The solutions are known and simply need to be implemented. Not only do we have an extraordinary opportunity to save tens of millions of lives in the decades ahead by reducing air pollution, we have a moral and legal obligation to do so.

*David R. Boyd is the United Nations Special Rapporteur on human rights and the environment, and an associate professor at the Institute for Resources, Environment and Sustainability at the University of British Columbia in Canada*.
